# Genetic Profiling of Sebaceous Carcinoma Arising from an Ovarian Mature Teratoma: A Case Report

**DOI:** 10.3390/ijms25126351

**Published:** 2024-06-08

**Authors:** Sumika Zaitsu, Yoko Aoyagi, Haruto Nishida, Kohei Nakamura, Mitsutake Yano, Eiji Kobayashi

**Affiliations:** 1 Department of Obstetrics and Gynecology, Faculty of Medicine, Oita University, Oita 879-5593, Japan; szaitsu@oita-u.ac.jp (S.Z.); yokoao@oita-u.ac.jp (Y.A.); ekobayashi@oita-u.ac.jp (E.K.); 2 Department of Diagnostic Pathology, Faculty of Medicine, Oita University, Oita 879-5593, Japan; nharuto@oita-u.ac.jp; 3 Genomics Unit, Keio Cancer Center, School of Medicine, Keio University, Tokyo 160-8582, Japan; knakamura320@keio.jp

**Keywords:** sebaceous carcinoma, ovarian teratoma, malignant transformation: endoreduplication, TP53, PIK3R1, uniparental disomy

## Abstract

Ovarian mature teratomas (OMTs) originate from post-meiotic germ cells. Malignant transformation occurs in approximately 1–2% of OMTs; however, sebaceous carcinoma arising from OMTs is rare. This is the first report of a detailed genomic analysis of sebaceous carcinoma arising from an OMT. A 36-year-old woman underwent evaluation for abdominal tumors and subsequent hysterectomy and salpingo-oophorectomy. Pathologically, a diagnosis of stage IA sebaceous carcinoma arising from an OMT was established. Eight months post-surgery, the patient was alive without recurrence. Immunohistochemically, the tumor was negative for mismatch repair proteins. A nonsense mutation in *TP53* (p.R306*) and a deletion in *PIK3R1* were identified. Single nucleotide polymorphisms across all chromosomes displayed a high degree of homozygosity, suggestive of uniparental disomy. Herein, the OMT resulting from the endoreduplication of oocytes underwent a malignant transformation to sebaceous carcinoma via *TP53* as an early event and *PIK3R1* as a late event.

## 1. Introduction

Ovarian mature teratomas (OMTs) originate from post-meiotic germ cells and are associated with genome-wide uniparental disomy (UPD) patterns [1,2]. As OMTs contain all three germ cell layers, and they often display multiple differentiated tissue types, including teeth, bone, and hair. Malignant transformation occurs in approximately 1–2% of OMTs, and the prognosis is poor at advanced stages [3]. Up to 80% of transformed teratomas contain squamous cell carcinoma, whereas, the remaining 20% contain adenocarcinoma, thyroid carcinoma, carcinoid tumors, and melanoma [3,4]. The malignant transformation of OMTs is characterized by difficulty in diagnosis and histological diversity [3,4,5,6].

Sebaceous carcinoma is a rare tumor with an incidence of 2.4 cases per million persons per year [7]. *TP53* (~70%), *RB1*, *ZNF750*, and *NOTCH1* have been identified as common genetic mutations [7]. Sebaceous carcinoma arising from an OMT is extremely rare, with only 14 previously reported cases [8,9,10,11,12,13,14,15,16,17,18,19,20]. Previous studies have reported the association between sebaceous carcinoma arising from OMTs and abnormalities in mismatch repair proteins/genes. However, genomic information is currently lacking. As sebaceous carcinomas originate from OMTs, it may have a unique genetic pattern related to meiosis [1,2]. Herein, we report a case of sebaceous carcinoma arising from an OMT and provide a detailed genomic analysis of the tumor.

## 2. Case Presentation

**Figure 1:**
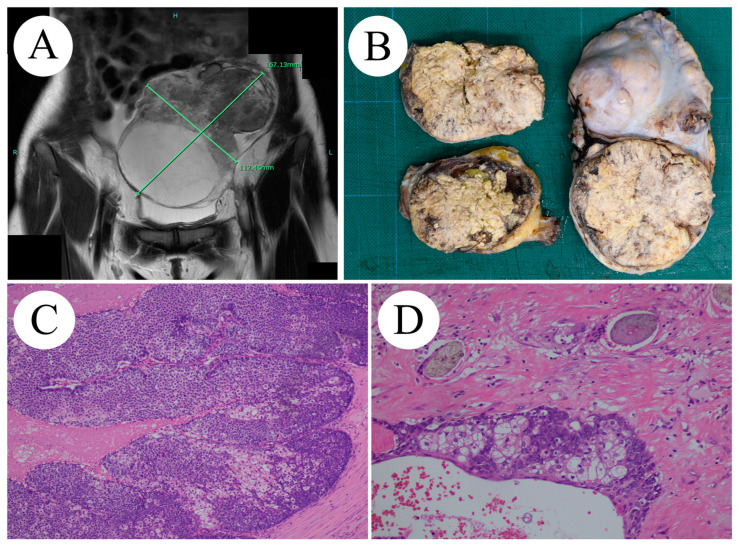
(**A**) MRI image findings: A 167 × 112 × 110 mm mass extending from the midline of the pelvis to the left with dissemination into the large omentum. (B) Macroscopic findings: solid proliferation mainly composed of fat components. (**C**,**D**) Histological findings (H&E staining): (**C**, ×10) proliferation of tumor cells with differentiation potential into sebaceous glands cells on the background of hairs, (**D**, ×40) proliferation of basophilic and mildly atypical cells with mitotic figure on the background of fibrosis and necrosis.

A 36-year-old woman (gravida 1, para 1) was referred to our hospital for an evaluation of abdominal tumors. Subsequently, she became pregnant. At the age of 27, she simultaneously underwent a right oophorectomy for an OMT and a cesarean section. She had no other relevant medical or family history. Her primary complaint was persistent abdominal swelling for 1 month. Systemic enhanced computed tomography and T2-weighted images from pelvic enhanced magnetic resonance imaging showed a 167 × 112 × 110 mm mass occupying the pelvic cavity, with suspected omental disseminations (Figure 1A). The preoperative diagnosis was stage IIIC left ovarian immature teratoma (cT3c cN0 M0). An intraoperative pathological diagnosis of sebaceous carcinoma was performed using frozen sections. The patient underwent total abdominal hysterectomy, left salpingo-oophorectomy, right salpingectomy, subtotal omentectomy, pelvic lymphadenectomy, and para-aortic lymphadenectomy. Complete resection was achieved. Macroscopically, the right ovarian tumor appeared as a well-circumscribed, yellowish-white mass with hair and partial necrosis (Figure 1B). Microscopically, the tumor exhibited sheets or lobules separated by a fibrovascular stroma of basophilic and atypical cells with central comedo-type necrosis (Figure 1C,D).

**Figure 2:**
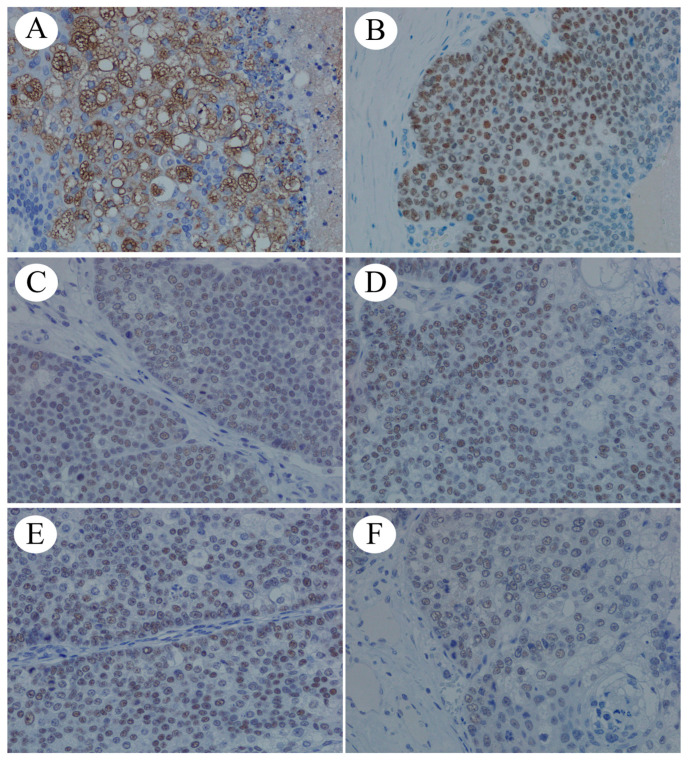
Immunohistochemical staining (×40): (**A**) androgen receptor, (**B**) adipophilin, (**C**) MLH1, (**D**) MSH2, (**E**) MSH6, and (**F**) PMS2.

The tumor exhibited an epidermis, hair follicles, and sebaceous glands. No dissemination to the greater omentum or lymph node metastases was observed. Immunohistochemical staining revealed tumor cells positive for androgen receptors (Figure 2A), GATA-binding protein 3, adipophilin (Figure 2B), and mismatch repair proteins MLH1 (Figure 2C), MSH2 (Figure 2D), MSH6 (Figure 2E), and PMS2 (Figure 2F).

Accordingly, a diagnosis of stage IA (pT1a, pN0, M0) sebaceous carcinoma arising from an OMT was established. No post-operative adjuvant therapy was administered. The patient was alive without recurrence 8 months post-operation.

**Figure 3:**
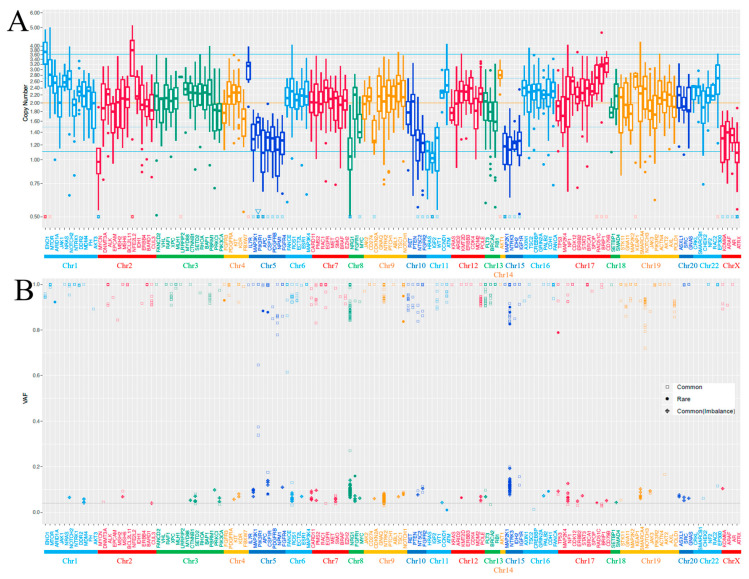
(**A**) Copy number alteration and (**B**) allelic frequencies of single nucleotide polymorphisms (SNPs) across all examined genes in a sebaceous carcinoma sample. The horizontal axis corresponds to each examined gene, and the vertical axis corresponds to the (**A**) copy number or (**B**) VAF. Common means >1% genetic alteration in HGVD (https://www.hgvd.genome.med.kyoto-u.ac.jp/ accessed on 14 September 2023), ToMMo (https://www.megabank.tohoku.ac.jp/english/ accessed on 14 September 2023), or gnomAD (https://gnomad.broadinstitute.org/ accessed on 14 September 2023), and rare means anything else.

Genomic DNA was obtained from a sample classified as a sebaceous carcinoma but not an OMT—which was considered a separate precursor lesion—because of limited sample volume. Cancer gene profiling was performed using the PleSSision system as previously described [21]. The average sequencing depth was 619.4× for sebaceous carcinoma. Histologically, the average tumor cellularity was 60%. In an analysis using a cancer gene panel of 160 genes in a sebaceous carcinoma sample, several actionable gene alterations were observed (Table S1). Nonsense mutation in *TP53* (p.R306*) and deletions in *PIK3R1*, *ATRX*, and *APC* were identified. In summary, a DNA quality check using the Agilent 2000 TapeStation (Agilent Technologies, Santa Clara, CA, USA), targeted amplicon exome sequencing using the Illumina MiSeq sequencing platform (Illumina, San Diego, CA, USA), and sequencing data analysis using the GenomeJack bioinformatics pipeline (version 1.0, Mitsubishi Space Software, Tokyo, Japan) were performed. The copy number loss/amplification cutoff was set to 1.1/3.6, which is statistically > 2σ. Single nucleotide polymorphisms across all chromosomes displayed a high degree of homozygosity, with allelic frequencies near 100% in nearly all of the genes examined (Figure 3 and Table S2). This pervasive pattern was evident across the entire genome. The tumor was characterized as microsatellite stable, and the tumor mutation burden was measured at 5.5 single nucleotide variants per megabase.

## 3. Discussion

To the best of our knowledge, this is the first report of a detailed genomic analysis of sebaceous carcinoma arising from an OMT. The 14 previously reported cases are summarized in Table 1 [8,9,10,11,12,13,14,15,16,17,18,19,20]. In the four reports that investigated mismatch repair abnormality, protein/gene abnormality was identified in all four cases [8,9,11,14]. However, since this case did not exhibit mismatch repair/gene abnormality and microsatellite instability, alternate pathogeneses had to be considered. Nonsense mutation of *TP53* (p.R306*) and a deletion in *PIK3R1* were detected in the targeted next-generation sequencing. Cooke et al. [22] reported that the most frequently altered genes were *TP53* (80%), *PIK3CA* (52%), and *CDKN2A* (44%), and that *TP53* mutation is an early event in squamous cell carcinoma arising from OMTs. Strikingly, 40% of the *TP53* mutations were biallelic, which may be associated with improved outcomes [22]. *KRAS* amplification and a deletion of *PTEN* and *RB1* were detected in malignant melanoma arising from OMT [21]. No previous studies have reported cases of OMT or sebaceous carcinoma with *PIK3R1* mutations. Rubinstein et al. [23] reported the safety and efficacy of the dual PI3K/mTOR inhibitor in patients with advanced endometrial cancer and activating mutations in the PI3K pathway, including *PIK3R1*. Single nucleotide polymorphisms across all chromosomes displayed a high degree of homozygosity, except *PIK3R1A*. *PIK3R1* mutations had a relatively low VAF because they were present in some, but not all, tumors. This *PIK3R1* mutation is considered a late event that occurs in a subclone after the malignant transformation of an OMT. The observed extensive homozygosity and the uniform allelic frequencies close to 100% suggest the presence of UPD across all chromosomes. Such widespread UPD is unusual and highlights a significant alteration from the expected heterozygous genetic landscape typical of diploid cells. The implications of this finding are profound, as UPD can lead to disruptions in gene expression, loss of heterozygosity, and potential impacts on tumor suppressor genes and oncogenes. This genetic uniformity may contribute to the oncogenic process by altering the cellular landscape, potentially leading to an enhanced tumorigenic capacity. The mechanisms driving such extensive UPD and its role in the progression of the tumor warrant further investigation to understand its contribution to cancer biology and therapy.

OMTs are classified into five types (I-V) based on their cytogenetic features [24]. Type I OMTs result from errors in meiosis I, type II OMTs result from meiosis II failure, type III OMTs occur via endoreduplication of a haploid ovum, type IV arises from oogonia, and type V OMTs are considered to originate from the fusion of two normal haploid ovaries [1,2,24]. Notably, the sebaceous carcinoma lacked heterozygous pleomorphism in the whole genome, suggesting that the sebaceous carcinoma arose from a type III OMT via endoreduplication of a haploid ovum [1,2]. Using short tandem repeat polymorphism analysis of centromeric and distal markers, Usui et al. reported that all OMTs were of post-meiotic origin rather than of pre-meiotic origin. Finally, the developmental process of this tumor is explained as follows. Type III OMTs resulted from the endoreduplication of oocytes after meioses I and II. As an early event, a *TP53* mutation occurred, leading to the development of sebaceous gland carcinoma. Subsequently, a subclone acquired a *PIK3R1* mutation as a late event. However, the present study is a case report, and future case studies are required to confirm the reproducibility of our findings.

In conclusion, an OMT resulting from the endoreduplication of oocytes underwent malignant transformation to sebaceous carcinoma via *TP53* as an early event and *PIK3R1* as a late event in the present case. These mutations may also be useful for prognosis prediction and targeted therapy. Genetic analysis is important to elucidate the pathogenesis of this rare tumor, and further case collection is required.

## Figures and Tables

**Table 1:** Summary of sebaceous carcinomas arising from mature ovarian teratomas.

Case Report (Year)	Age	Medical History	Stage	Treatment	IHC	IHC of MMR	**Genetic Analysis**	**Reference**
The present case	36	None	IA	TAH+BSO+OM+LA without AC	Positive for androgen receptor, GATA-binding protein 3, and adipophilin Aberrant expression for p53 (null type)	Positive for MLH1, MSH2, MSH6, and PMS2	Nonsense mutation in TP53, deletion in PIK3R1, ATRX, and APC, and microsatellite stable	NA
Mohammed M (2023)	49	NA	1C	TAH+BSO	NA	Loss of MSH2 and MSH6	NA	[8]
Murray J (2022)	49	NA	IC2	TAH+BSO without AC	Diffuse positive for adipophilin and CK5/6; negative for CK7	Loss of MSH2 and MSH6; positive for MLH1 and PMS2	Nonsense variant c.1102G > T [p.(Glu368*)] in exon 7 of MSH2	[9]
de Lima RB (2018)	59	NA	NA	TAH+BSO+OM (secondary surgery)	NA	NA	NA	[10]
Wield A (2018)	67	Basal cell carcinoma, seborrheic dermatitis, actinic keratosis	IC	TLH+BSO+OM+LA without AC	Overexpression of p53 and scattered expression of p16	Loss of MSH2 and MSH6	Somatic inactivation of MSH2 and MSH6	[11]
Moghaddam Y (2013)	66	NA	NA	TAH+BSO+OM+LA without AC	Positive for CK5, p63, and androgen receptor	NA	NA	[12]
An HJ (2013)	69	NA	IIIB	TAH+BSO+OM	Positive for CK7; negative for CK20 and p53	NA	NA	[13]
Smith J (2012)	52	OMT, basal cell carcinomas, keratoacanthoma, intraepidermal carcinomas, ductal carcinoma, sebaceous adenoma	NA	TAH+BSO	NA	Loss of MSH2 and MSH6; positive for MLH1 and PMS2	NA	[14]
Venizelos ID (2009)	74	NA	IA	TAH+BSO+OM	Positive for CK7 and overexpression of p53	NA	NA	[15]
Ribeiro-Silva A (2003)	63	None	NA	RSO	Positive for CK7 and no overexpression of p53	NA	NA	[16]
Vartanian RK (2002)	39	NA	IA	TAH+BSO	NA	NA	NA	[17]
Changchien CC (1994)	64	Diabetic mellites, hypertension	NA	TAH+BSO with AC	NA	NA	NA	[18]
Chumas JC (1991)	31	NA	NA	TAH+BSO+OM without AC	NA	NA	NA	[19]
Betta PG (1984)	53	NA	NA	TAH+BSO+OM	NA	NA	NA	[20]

IHC, immunohistochemistry; MMR, mismatch repair; TAH, total abdominal hysterectomy; TLH, total laparoscopic hysterectomy; BSO, bilateral salpingo-oophorectomy; RSO, right salpingo-oophorectomy; OM, omentectomy; LA, lymphadenectomy; AC, adjuvant chemotherapy; NA, not available.

## Data Availability

The datasets used and/or analyzed during the current study are available from the corresponding author upon reasonable request.

## Supplementary Materials

The following supporting information can be downloaded at: https://www.mdpi.com/article/10.3390/ijms25126351/s1.
