# Supplementary material for: Genetic Profiling of Sebaceous Carcinoma Arising from an Ovarian Mature Teratoma: A Case Report
Source: Int J Mol Sci. 2024 Jun 8;25(12):6351. doi: 10.3390/ijms25126351 (PMC11203495; doi:10.3390/ijms25126351)
Supplement: Supplementary file 1 [file ijms-25-06351-s001.zip › ijms-3029491-supplementary.pdf]

Table S1. Summary of the genetic alteration in the present case.

Gene	Alteration	Variant allele frequency (%)
ATRX	Loss	Loss
BARD1	p.V507M	99.8
ROS1	p.T776M	5.8
SETBP1	c.540+7405_540+7422	90.6
TP53	p.R306*	78.8
PIK3R1	Loss	Loss
APC	Loss	Loss
KDM6A	c.335-70C>A	100
NOTCH1	p.T1379M	8.5

Table S2. Summary of the single nucleotide polymorphisms in the present case.

Gene	Single nucleotide polymorphism	Variant allele frequency (%)	Chromosome
BARD1	c.1904-413G>A	100.0%	chr2
BARD1	c.1904-533G>C	100.0%	chr2
BARD1	c.1568+78_1568+79delAA	100.0%	chr2
BARD1	c.1568+51A>G	100.0%	chr2
BARD1	c.1568+14C>T	100.0%	chr2
BARD1	c.1518_1519delinsCA	99.8%	chr2
BARD1	c.1315-19G>A	99.8%	chr2
BARD1	c.1134G>C	99.9%	chr2
BARD1	c.158+46A>C	100.0%	chr2
BARD1	c.70C>T	100.0%	chr2
BARD1	c.-30G>C	100.0%	chr2
BARD1	c.-48T>C	100.0%	chr2
BARD1	c.-78G>A	100.0%	chr2
PIK3R1	c.219C>T	100.0%	chr5
PIK3R1	c.334+14A>C	100.0%	chr5
PIK3R1	c.427+81A>C	64.7%	chr5
PIK3R1	c.428-21G>A	33.8%	chr5
PIK3R1	c.978G>A	37.4%	chr5
APC	c.730-71_730-70insT	88.4%	chr5
APC	c.1458T>C	100.0%	chr5
APC	c.1635G>A	99.4%	chr5
APC	c.4479G>A	99.7%	chr5

APC	c.5034G>A	100.0%	chr5
APC	c.5268T>G	99.7%	chr5
APC	c.5465T>A	99.4%	chr5
APC	c.5880G>A	100.0%	chr5
ROS1	c.6720T>G	5.3%	chr6
ROS1	c.5704G>A	4.9%	chr6
ROS1	c.5642-1437C>T	5.9%	chr6
ROS1	c.5641+629A>G	6.5%	chr6
ROS1	c.5557+615A>G	5.6%	chr6
ROS1	c.5557+93_5557+136delTGACTATATATATATATATATATATATATATATATATAATCT	44.0%	chr6
ROS1	c.5367-12_5367-11insT	6.9%	chr6
ROS1	c.5249-191C>T	13.2%	chr6
ROS1	c.5249-579C>T	6.0%	chr6
ROS1	c.5249-1317A>G	8.5%	chr6
ROS1	c.5248+1996C>T	7.7%	chr6
ROS1	c.5248+1780A>C	100.0%	chr6
ROS1	c.5248+1433C>G	7.0%	chr6
ROS1	c.3855-5T>C	6.0%	chr6
ROS1	c.3445+41_3445+42insGTGT	46.7%	chr6
ROS1	c.2973+45A>G	94.5%	chr6
ROS1	c.2813-39T>A	95.3%	chr6
ROS1	c.2327C>T	5.8%	chr6
ROS1	c.1262+41G>C	94.0%	chr6
ROS1	c.439-22G>T	94.4%	chr6
ROS1	c.439-59T>G	98.2%	chr6

ROS1	c.438+45delT	18.0%	chr6
ROS1	c.303A>T	92.3%	chr6
ROS1	c.229-14C>T	94.9%	chr6
ROS1	c.228+1694delT	9.4%	chr6
ROS1	c.228+31C>T	92.3%	chr6
NOTCH1	c.6555C>T	100.0%	chr9
NOTCH1	c.6180+5G>A	83.7%	chr9
NOTCH1	c.5473-43T>C	99.4%	chr9
NOTCH1	c.5384+34G>A	8.9%	chr9
NOTCH1	c.5094C>T	100.0%	chr9
NOTCH1	c.4136C>T	8.5%	chr9
NOTCH1	c.4014+75G>A	100.0%	chr9
NOTCH1	c.3644-79C>T	100.0%	chr9
NOTCH1	c.3325+21A>G	100.0%	chr9
NOTCH1	c.3171+54A>G	100.0%	chr9
NOTCH1	c.2970-31A>G	100.0%	chr9
NOTCH1	c.2868C>T	7.7%	chr9
NOTCH1	c.2588-4G>A	100.0%	chr9
NOTCH1	c.2587+103T>C	100.0%	chr9
NOTCH1	c.2467+21G>A	100.0%	chr9
NOTCH1	c.2265T>C	99.8%	chr9
NOTCH1	c.2015-30G>T	95.0%	chr9
NOTCH1	c.1670-9A>G	100.0%	chr9
NOTCH1	c.1669+124_1669+131delCTCAGCCC	100.0%	chr9
NOTCH1	c.1669+9T>C	100.0%	chr9

NOTCH1	c.1556-43T>C	100.0%	chr9
NOTCH1	c.1555+10A>G	100.0%	chr9
NOTCH1	c.1442-43C>T	100.0%	chr9
NOTCH1	c.1441+7C>T	100.0%	chr9
NOTCH1	c.312T>C	99.6%	chr9
TP53	c.916C>T	78.8%	chr17
TP53	c.782+72C>T	92.7%	chr17
TP53	c.672+62A>G	100.0%	chr17
TP53	c.376-91G>A	100.0%	chr17
TP53	c.215C>G	9.3%	chr17
TP53	c.96+16_96+31delGGGCTGGGGACCTGGA	68.9%	chr17
SETBP1	c.540+7405_540+7422delinsACAAAACCCGGTTCTCTCTCTT	90.6%	chr18
SETBP1	c.3825A>G	99.8%	chr18
KDM6A	c.335-70C>A	100.0%	chrX
KDM6A	c.619+30A>G	91.4%	chrX
KDM6A	c.2177C>A	89.2%	chrX
KDM6A	c.2703-6_2703-5delTT	88.5%	chrX
KDM6A	c.3111G>A	10.4%	chrX
KDM6A	c.3737-6delT	10.5%	chrX
